# Blueberry Anthocyanins-Enriched Extracts Attenuate Cyclophosphamide-Induced Cardiac Injury

**DOI:** 10.1371/journal.pone.0127813

**Published:** 2015-07-02

**Authors:** Yunen Liu, Dehong Tan, Lin Shi, Xinwei Liu, Yubiao Zhang, Changci Tong, Dequn Song, Mingxiao Hou

**Affiliations:** 1 Emergency Medicine Department of General Hospital of Shenyang Military Command, Laboratory of Rescue Center of Severe Wound and Trauma PLA, Shenyang, China; 2 College of Food, Shenyang Agricultural University, Shenyang, China; University of Hawai'i Manoa, UNITED STATES

## Abstract

We sought to explore the effect of blueberry anthocyanins-enriched extracts (BAE) on cyclophosphamide (CTX)-induced cardiac injury. The rats were divided randomly into five groups including normal control, CTX 100 mg/kg, BAE 80mg/kg, CTX+BAE 20mg/kg and CTX+BAE 80mg/kg groups. The rats in the three BAE-treated groups were administered BAE for four weeks. Seven days after BAE administration, rats in CTX group and two BAE-treated groups were intraperitoneally injected with a single dose of 100 mg/kg CTX. Cardiac injury was assessed using physiological parameters, Echo, morphological staining, real-time PCR and western blot. In addition, cardiotoxicity indices, inflammatory cytokines expression and oxidative stress markers were also detected. Four weeks 20mg/kg and 80mg/kg dose of BAE treatment following CTX exposure attenuated mean arterial blood pressure, heart rate and activities of heart enzymes, improved cardiac dysfunction, left ventricular hypertrophy and fibrosis. Importantly, BAE also attenuated CTX-induced LV leukocyte infiltration and inflammatory cytokines expression, ameliorated oxidative stress as well as cardiomyocyte apoptosis. In conclusion, BAE attenuated the CTX-induced cardiac injury and the protective mechanisms were related closely to the anti-inflammatory, antioxidant and anti-inflammatory characteristics of BAE.

## Introduction

The cyclophosphamide (CTX) which is a commonly used alkylating agent, has been used widely from non-neoplastic diseases to neoplastic diseases as an immunosuppressive agent [1]. The CTX, activated by hepatic cytochrome P450 oxidase system,was decomposed into phosphoramide mustard and acrolein [2]. Phosphoramide mustard is linked to mostly CTX’s therapeutic effects, while acrolein is associated with the side effects. Acrolein interferes with the tissue antioxidant defense system and induces ROS, which causes cardiac injury, arrhythmias and congestive heart failure [3–6]. Myocardial injury is one of the most serious side effect of CTX, which usually leads to the death of cancer patients. It is well documented that the CTX-induced acute cardiotoxicity was associated with the disorder of the antioxidant defense system. Oxidative stress, exerted both agonistic and antagonistic effect, has been widely used to regulate apoptotic signaling [7,8]. Thus how to attenuate CTX-induced cardiotoxicity is important significance.

It is well-known that blueberry (Vaccinium corymbosum L.) is recognized as a good source of anthocyanins [9]. The anthocyanins extracted in blueberries are 3-glycosidic derivatives of cyanidin, delphinidin, malvidin, petunidin, and peonidin [10]. A lot of studies previous have shown that blueberry anthocyanins activated cellular antioxidant system, and inhibited inflammatory gene expression, consequently protected against oxidant or inflammatory-induced organs toxicity [11–13], with potential health benefits including cardiovascular protection, neuroprotection, anticarcinogen, and antidiabetes [14,15]. However, it is not known whether blueberry anthocyanins-enriched extracts (BAE) have an effect on CTX-induced cardiac hypertrophy and injury. We hypothesized that BAE may protect against cardiac hypertrophy and injury. To test this idea, we investigated the effects of BAE on cardiac hypertrophy and injury induced by CTX exposure, and attempted to find a possible mechanism of action.

## Materials and Methods

### Experimental animals

The animal experiment was compliant with World Medical Association Declaration of Helsinki, and approved by the Animal Ethics Committee of General Hospital of Shenyang Military Command. Health male Sprague-Dawley (SD) rats (220 ± 20 g) were obtained from Liaoning Provincial Laboratory Animal Public Service Center (China, Benxi city). Animals were acclimatized to the experimental conditions for a period of 1 week before the initiation of the experiment. Rats were housed at 22 ± 2°C under a 12-h light—dark cycle, accessed to standard laboratory animal feed and water ad libitum.

### Materials and reagents

Blueberry anthocyanins-enriched extracts (BAE) preparation: according to the previous reports [16], blueberry anthocyanins were extracted from Saintcloud blueberry and purified by macroporous resins, lyophilized, powdered and preserved at –20°C for use. The total anthocyanin content was about 25.7g/100g, determined using the pH differential method [17]. The main anthocyanins were malvidin 3-galactoside (28.11%), malvidin 3-arabinoside (16.18%), malvidin 3-glucoside (14.08%), malvidin 3-(6''-acetyl) glucoside (8.49%), malvidin 3-(6''-acetyl) galacto-side (5.50%), petunidin 3-galactoside (5.44%), petunidin 3-glucoside (5.26%), peonidin3-glucoside (5.22%), cyanidin3-galactoside (2.96%), and delphinidin 3-glucoside (1.41%), as measured by HPLC/MS.

### Experimental protocol

After acclimation, the rats were divided randomly into five groups—normal control, BAE 80mg/kg, CTX 100mg/kg, CTX+ BAE 20mg/kg and CTX+ BAE 80mg/kg groups, each containing eight rats. The rats in the two BAE-treated groups were administered by gavage (distilled water as normal) with BAE 20 and 80 mg/kg per day, respectively, for four weeks, while the rats in the other two groups were given the same volume of distilled water. Seven days after BAE administration, rats in CTX and BAE-treated groups were intraperitoneally (normal saline as normal) injected with a single dose of 100 mg/kg CTX (Hengrui pharmaceutical Co. Ltd, China). Rats in the normal treated group were given equal volume of normal saline.

### Echo

All aimals under light anaesthesia were conducted by Echo (Vevo 770, a 12 MHz transducer) refer to the previous literature [18]. Briefly, to ensure that the mitral and aortic valves and the apex were visualized, parasternal long axis views were obtained and recorded and short axis views were recorded at the mid-papillary muscle level. In order to calculate end diastolic and end systolic LV area, endocardial area tracings were conducted in 2D mode from digital images captured on cineloop. All measurements were made by a single observer and were averaged over three to five consecutive cardiac cycles. The reproducibility of measurements was assessed in two sets of baseline measurements in 10 randomly selected rats, and the repeated measure variability did not exceed 65%.

### Blood pressure monitoring

Blood pressure monitoring was done as described previously [19]. Briefly, All rats were anaesthetized with phenobarbital sodium (i.p, 50 mg/kg) and placed on a servocontrolled heating pad maintaining body temperature at 37°C. Blood pressure was measured with a fiberoptic transducer inserted in the left carotid artery. After 15 minutes of stabilization, the pressure was continuously sampled for 10 minutes for later analysis.

### Sample collection

Twenty four hours after the last BAE treatment, rats were anesthetized with phenobarbital sodium (i.p, 50 mg/kg) and blood samples were obtained from abdominal aorta. Sera were collected and stored for biochemical analysis. Tissues for protein analysis were freshly frozen in liquid nitrogen, weighted on an electronic balance and stored in liquid nitrogen until transfer into a -80°C freezer. All samples were stored in a -80°C freezer until used.

### Biochemical analysis

Activities of aspartate aminotransferase (AST) and lactate dehydrogenase (LDH) in plasma were detected by the methods of Buhl and Jackson [20]. The creatine kinase (CK-MB) activity was determined according to the method described by Wu and Bowers et al [21].

### Histological analysis

Samples for histological analysis were fixed in 10% formaldehyde at room temperature and embedded in paraffin blocks using a Leica Microsystem tissue processor (ASP 300S, Germany). For histological staining, sections of 3 μm thickness were sliced using a Leica Microsystem microtome (Model RM 2265, Germany). LV fibrosis was stained using modified masson’s trichrome stain Kit (Sigma, America), and hematoxylin-eosin staining (H&E) was performed.

### Immunohistochemistry and immunofluorescence

Immunostaining was conducted to identify the cellular expression of mouse anti-rat CD45,Bcl-2 and bax using a standard immunostaining kit (Sigma, USA). Antibodies against CD45,Bcl-2 and Bax were purchased from Abcam (Abcam, USA) and used at 1:50 dilution. After incubation with primary antibodies, the sections were washed with PBS and incubated with the secondary antibody (Sigma, USA). Immunohistochemistry visualization was completed using 3,3-diaminobenzidin (DAB) (Sigma, USA) and counterstained with hematoxylin solution. Negative control was included and performed the same immunohistochemical method with omission of the primary antibodies.

### Real-time PCR

Total RNA was extracted from heart tissue with TRIzol (Takara Biotechnology, Tokyo, Japan) and reverse-transcribed using a complementary DNA reverse transcription kit (Invitrogen, Carlsbad, CA, USA). Reactions were performed in a real-time PCR thermocycler (Bio-Rad, Hercules, CA, USA) using SYBR green as the fluorescence dye. The mRNA expression of the target genes was normalized to the control glyceraldehyde-3-phosphate dehydrogenase (GAPDH) using the comparative threshold cycle method. Real-time PCR was performed by using SYBR Green PCR

Master Mix (2X) (Takara, Takara Bio Inc, Japan) and following gene specific primers: forward primer, 5′-AGAGCAAAAGCAAAGGGTTTC-3′ and reverse primer, 5′-GTGATGGTACGAGATGGGCTA-3′ for β-MHC; forward primer, 5′- AGCGAGCA GACCGATGAAG-3′ and reverse primer, 5′-AGCCCTCAGTTTG CTTTTCA-3′ for ANP; forward primer, 5′-TGATTCTG CTCCTGCTTTC-3′ and reverse primer, 5′-GTGGATTGTTCTGGAG ACTG-3′ for BNP.

### Enzyme-linked immunosorbent assay

The levels of TNF-α, IL-1β, IL-10 and TLR4 in heart tissues were measured by enzyme-linked immunosorbent assay (ELISA), using commercially available kits (Zhong Shan Golden Bridge Biological Technology Co, Beijing, China) according to the manufacturer's instructions.

### Oxidative stress level determination

The levels of the oxidative stress marker including malondialdehyde (MDA), superoxide dismutase (SOD) and glutathione (GSH) in cardiac tissues were detected. MDA level was determined as described previously [22]. Superoxide dismutase (SOD) activity was measured according to the photochemical method [23] and GSH level were assayed spectrophotometrically at 412 nm and the contents of GSH were expressed as μmol/gm wet tissue [24].

### Western blot

Western blot was done as described previously [25]. Briefly, approximately 20 μg (20 μl) protein per gel well was loaded and resolved by 10% sodium dodecyl sulfate polyacrylamide gel electrophoresis(SDS-PAGE). The SDS-PAGE gel was transferred to polyvinylidene difluoride membranes. The membranes were incubated in tris-buffered saline (T-TBS) containing 3% non-fat dry milk and a specific proportion of the primary antibodies for bax and bcl-2(Cell signaling Technology) overnight at 4°C. The blot was then washed and incubated with goat anti-mouse IgG conjugated to peroxidase (Origene, America). Antibody binding was detected by chemoluminescence staining using the ECL detection kit (Bio-Rad). The density of each band was quantified by densitometry of Bandscan 5.0 software.

### Statistical analysis

Data were statistically treated by analysis of variance using SPSS (IBM, Armonk, NY, USA), followed by Student Newman Keul’s post-hoc test. The data were expressed as mean ± standard deviation. p<0.05 was considered statistically significant.

## Results

### BAE attenuated CTX-induced physiological parameters

**Fig 1:**
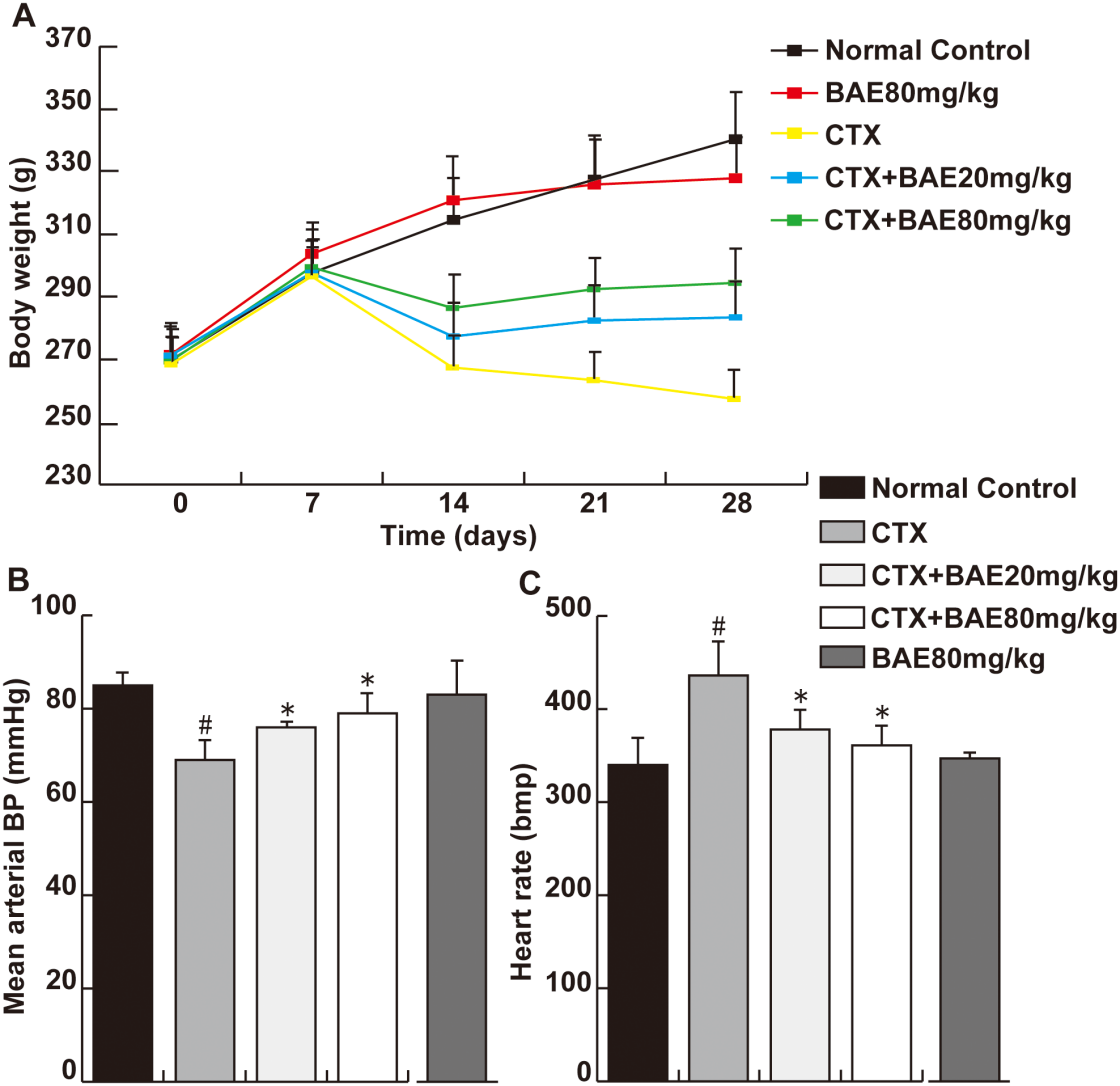
BAE attenuated CTX-induced physiological parameters. (A): Body weight. (B): Mean arterial BP. (C): Heart rate. Data are mean ± SD. ^#^ p < 0.05, compared to normal control group; * p < 0.05, compared to CTX.

At the end of study, the rats in normal control group and BAE 80 mg/kg group were in a good state with normal breathing, quick reflexes, and healthy looking fur. Whereas weakness, bloody urine, shortness of breath and diarrhea were found in the CTX group. These appearances were somewhat alleviated in both BAE groups. The body weight was decreased significantly in the CTX group compared with normal control group. Both BAE group animals had a significant increased in the body weight compared to CTX group (Fig 1A, p< 0.05). In addition, the CTX treated animals had a significantly decreased mean arterial blood pressure and increased heart rate compared to controls, which was improved significantly in the both BAE groups (Fig 1B and 1C, p< 0.05).

### BAE attenuated CTX-induced cardiac hypertrophy

**Fig 2:**
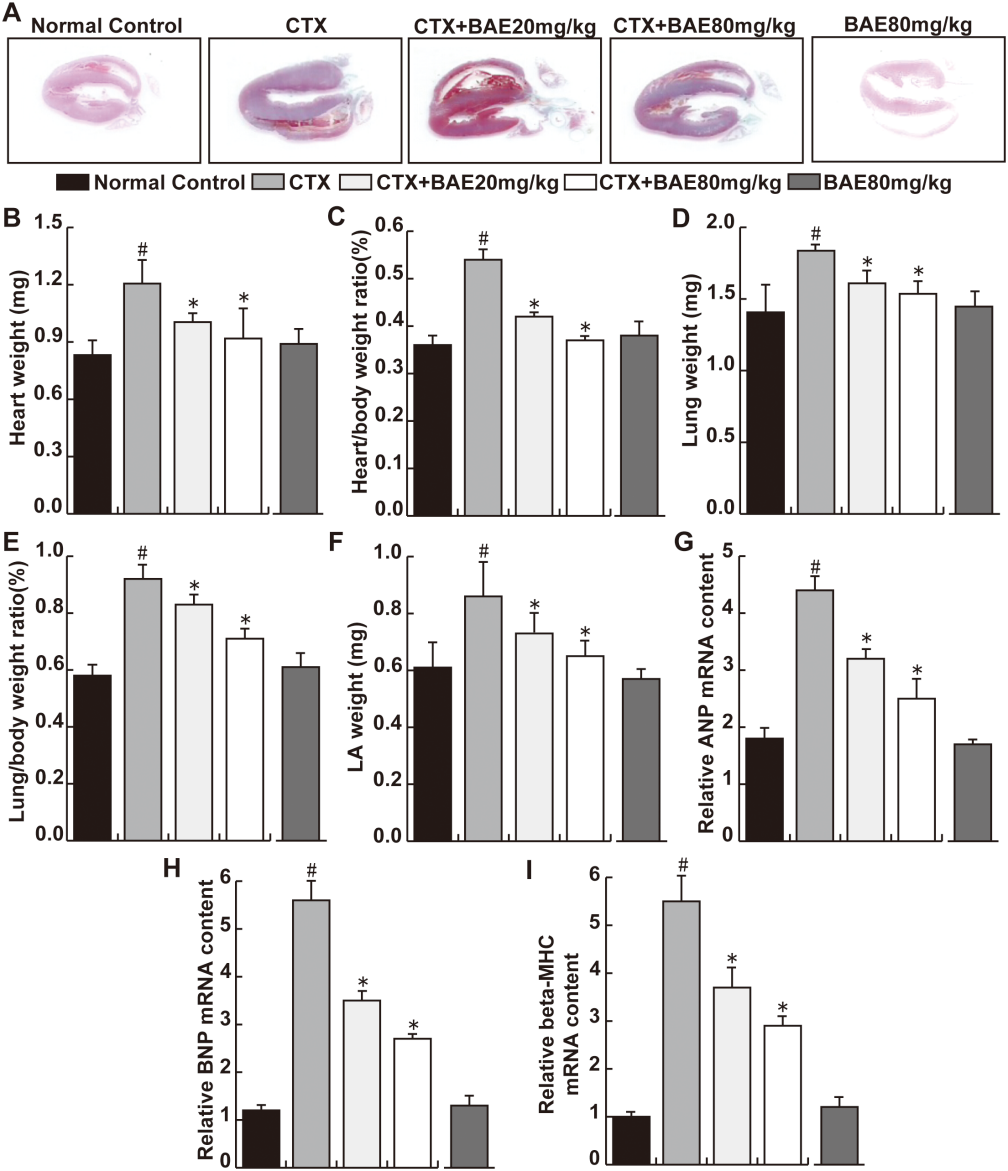
BAE attenuated CTX-induced cardiac hypertrophy. (A)Representative photographs of hearts at four weeks after CTX exposure. (B) Heart weight (mg). (C) Heart /body weight ratio. (D) Lung weight (mg). (E) Lung /body weight ratio. (F) LA weight (mg). Real time PCR of relative mRNA levels of (G) ANP, (H) BNP and (I) beta-MHC. Data are mean ± SD. ^#^ p < 0.05, compared to normal control group; * p < 0.05, compared to CTX.

Under control conditions, BAE had no detectable effect on the ratios of heart weight, lung weight, left atrial weight and their ratios to bodyweight (Fig 2). CTX cause significant increases of the heart weight, heart /body weight ratio, lung/body weight ratio and LA weight, while above changes were significantly abolished by BAE treatments (Fig 2A–2F, p< 0.05). In addition, CTX caused significant increases of mRNA content of LV atrial natriuretic peptide (ANP), brain natriuretic peptide (BNP) and myosin heavy chain (β-MHC). BAE significantly attenuated CTX-induced increases of mRNA content of ANP, BNP and β-MHC(Fig 2G–2I, p< 0.05).

### BAE attenuated CTX-induced LV dysfunction

Under control conditions, BAE had no detectable effect on LV ejection fractional shortening, LV ejection fraction, LV dimensions and LV wall thicknesses (Table 1). CTX caused significant decreases in LV fractional shorting and LV ejection fraction. CTX also caused significant increases in LV end-systolic, end-diastolic diameters, and LV wall thickness (Table 1). BAE significantly attenuated the CTX-induced decreases of LV ejection fraction and LV fractional shorting, and increases of LV end-diastolic and end-systolic diameters (Table 1).

**Table 1:** BAE attenuated CTX-induced cardiac dysfunction ($\bar{x}$±s).

Parameters	Normal control	CTX	CTX+BAE20mg/kg	CTX+BAE80mg/kg	BAE80mg/kg
LVPW(mm)	1.39±0.16	2.01±0.25^#^	1.71±0.08*	1.42±0.02*	1.33±0.12
LVEDd(mm)	4.95±0.53	6.91±0.47^#^	5.41±0.32*	5.24±0.58*	5.01±0.74
LVESd(mm)	3.12±0.47	5.765±0.71^#^	4.08±0.53*	3.64±0.36*	3.08±0.38
LVEDV(ml)	0.25±0.04	0.54±0.03^#^	0.45±0.03*	0.36±0.06*	0.24±0.02
LVESV(ml)	0.05±0.01	0.34±0.03^#^	0.13±0.05*	0.07±0.03*	0.06±0.02
LVEF(%)	62.32±5.95	31.42±3.18^#^	51.34±0.34*	56.56±0.53*	63.16±6.02
LVFS(%)	32.64±4.42	19.65±3.08^#^	27.32±1.38*	30.49±1.38*	33.83±4.15

^#^ p < 0.05, compared to normal control group

* p < 0.05, compared to CTX

### BAE attenuated CTX-induced increases of plasma AST, LDH and CK-MB

**Fig 3:**
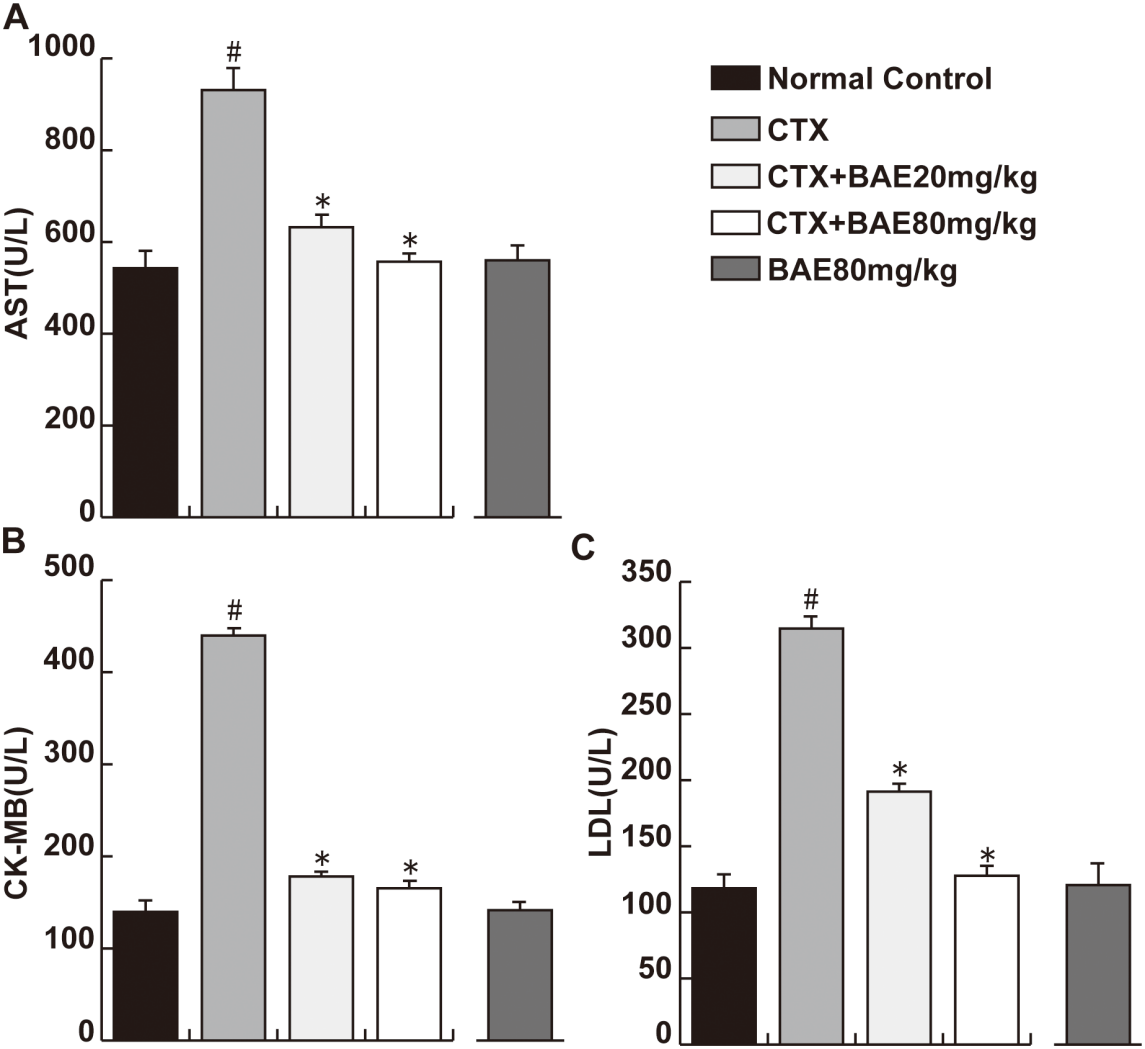
BAE attenuated CTX-induced increases of cardiotoxicity indices. (A) plasma AST level, (B) plasma CK-MB level, (C) plasma LDH level. Data are mean ± SD. ^#^p< 0.05, compared to normal control; *p < 0.05, compared to CTX.

CTX-induced cardiotoxicity was clearly observed by the increase in plasma cardiotoxicity indices. Activities of cardiotoxicity indices AST, LDH and CK-MB were significantly increased in the CTX group, respectively, compared to the normal group. Daily administration of 20 and 80 mg/kg BAE group to CTX-treated rats resulted in a obvious reversal of CTX-induced increase in AST, CK-MB and LDH to the normal values(Fig 3A–3C, p< 0.05).

### BAE attenuated CTX-induced LV fibrosis

**Fig 4:**
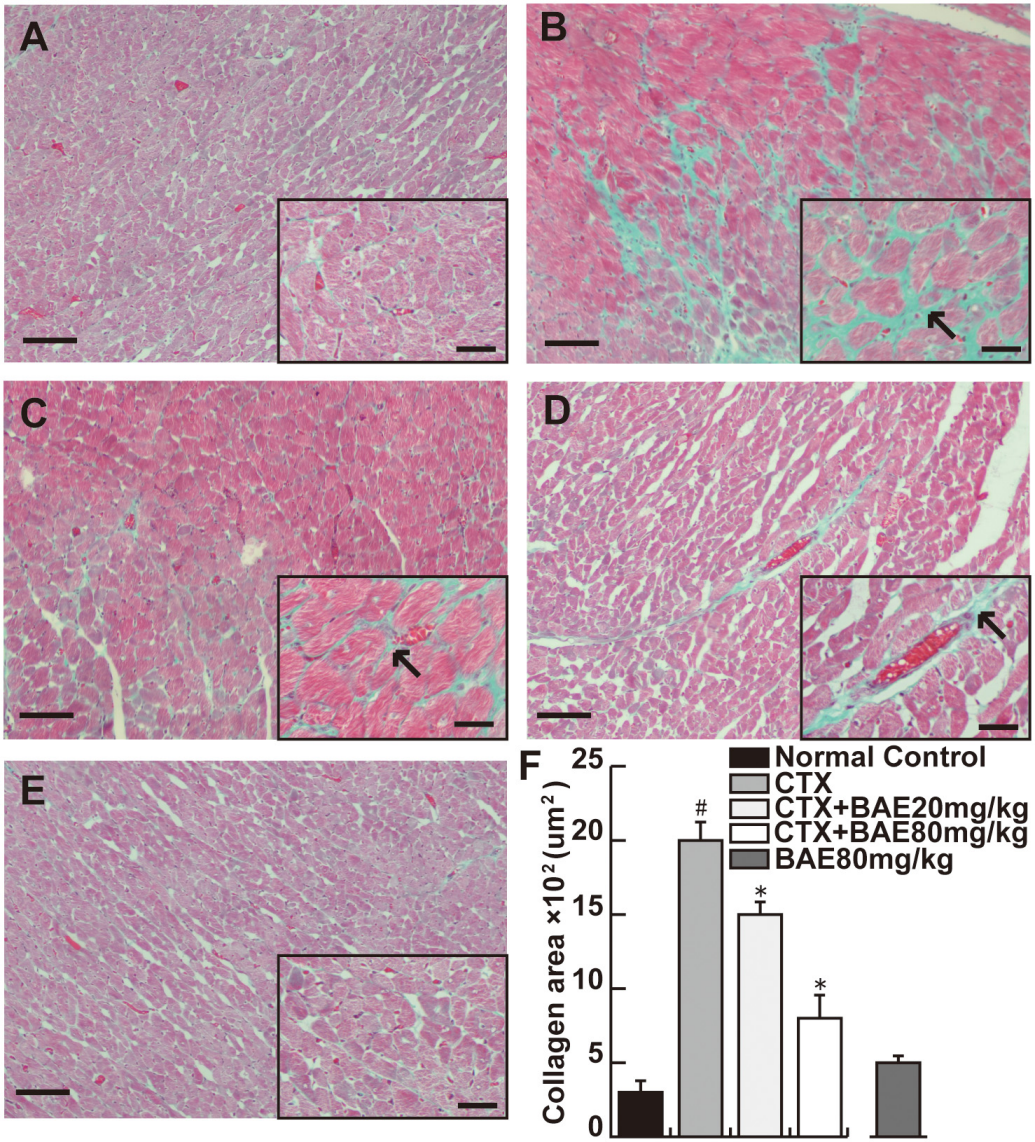
BAE attenuated CTX-induced LV fibrosis. Masson trichrome—stained sections of left ventricles. (A) Normal control; (B) CTX group; (C) CTX+BAE 20mg/kg group; (D) CTX+BAE 80mg/kg group; (E) BAE80mg/kg. The arrow represents the cardiac fibrosis (Scale bar: 20μm). (F) Quantification of cardiac fibrosis area from Masson trichrome—stained sections. Data are mean ± SD. ^#^ p < 0.05, compared to normal control; *p < 0.05, compared to CTX.

Masson trichrome staining show that CTX caused significant increase of LV fibrosis. BAE significantly attenuated CTX-induced increase of LV fibrosis (Fig 4A–4F, p< 0.05).

### BAE attenuated CTX-induced LV inflammation

**Fig 5:**
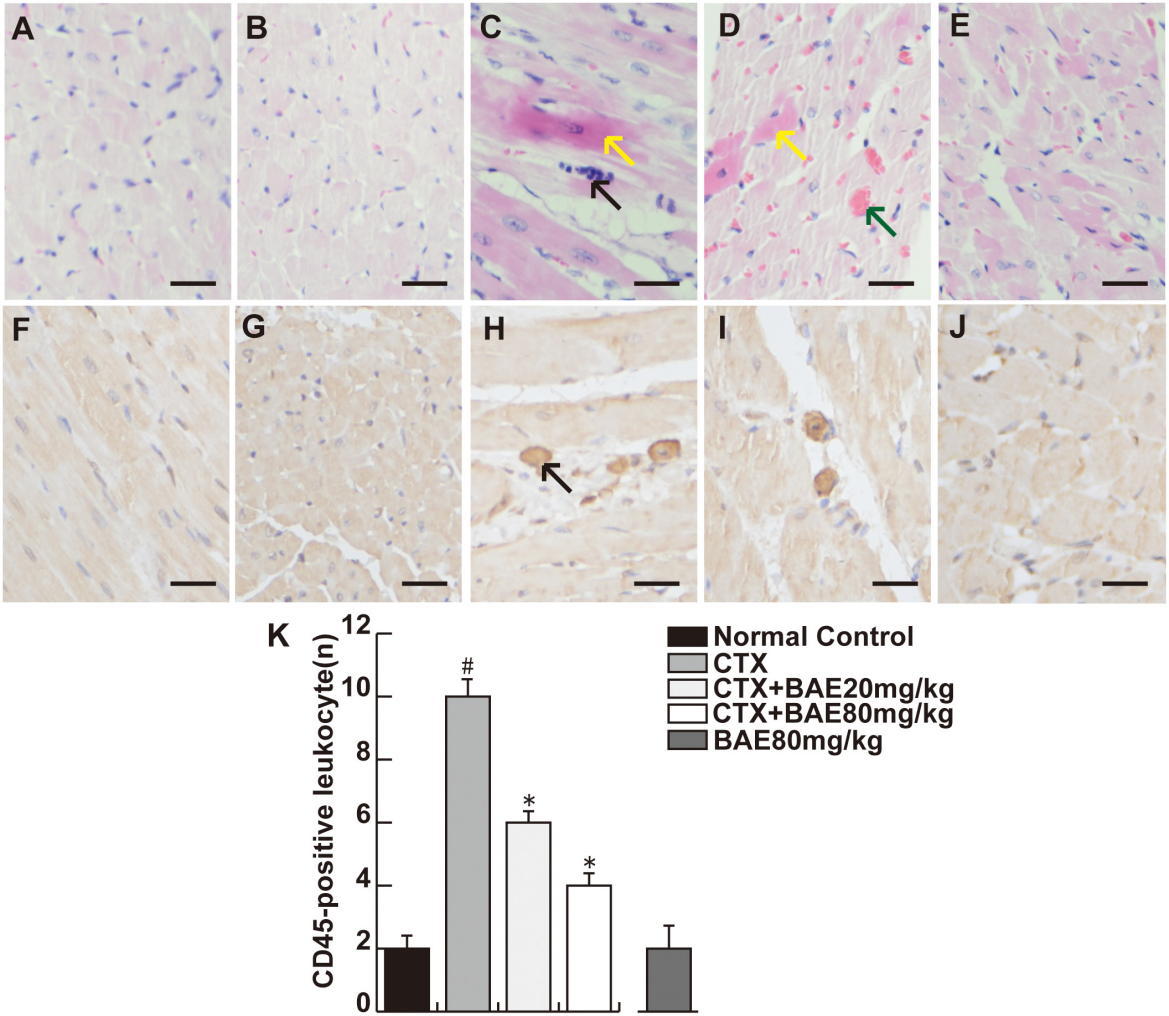
BAE attenuated CTX-induced LV inflammation. A and F: Normal control; B and G: BAE80mg/kg; C and H: CTX group; D and I: CTX+BAE20mg/kg; E and J: CTX+BAE80mg/kg. (A-E) show the histopathological changes in CTX-induced cardiomyocyte. The black arrow in C and D represents respectively inflammatory cells infiltration and heart congestion, whereas the brown arrow represents cardiomyocyte apoptosis (HE staining, Bar: 20 μm). (F-J) demonstrate the infiltration of CD45-positive leukocytes in the left ventricle of the rats in four groups. The arrow in H represents the CD45-positive leukocytes (immunohistochemical staining, Bar: 20 μm). (K) Bar charts show a number of CD45-positive leukocytes in the myocardial interstitium. Data are mean ± SD. ^#^p < 0.05, compared to normal control group; *p < 0.05, compared to CTX.

No heart injury was observed in normal control group animals. In contrast, extensive heart injury was observed in animals from the CTX group, there was obvious cardiac hypertrophy, heart edema with inflammatory cell infiltration. BAE attenuated the histopathological changes induced by CTX, especially in the 80 mg/kg group, with less myocardial leukocyte infiltration (Fig 5A–5E). To assess the effects of BAE on myocardial leukocyte infiltration, we investigated LV leukocyte infiltration density using CD45 immunohistochemical staining. There was a significant increase in the number of CD45-positive in CTX group compared with in the normal control group. However, there was significant decreased of leukocyte infiltration density in the BAE 20mg/kg and BAE80mg/kg groups compared with the CTX group (Fig 5F–5K, p< 0.05).

### BAE attenuated CTX-induced increase of pro-inflammatory cytokines

**Fig 6:**
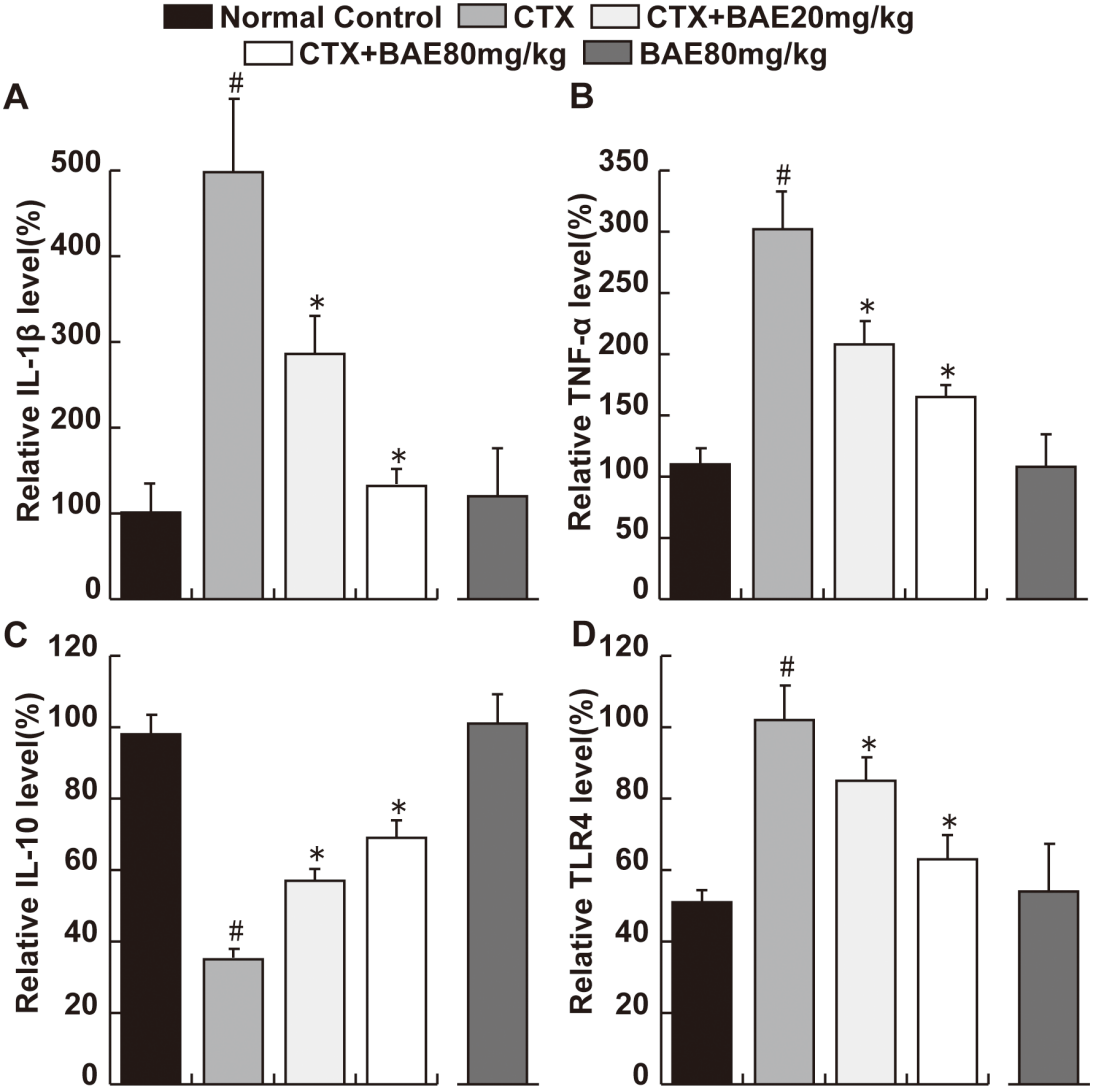
BAE attenuated CTX-induced increase of pro-inflammatory cytokines. ELISA assays were performed to detect the levels of IL-1β(A), TNF-α (B), IL-10 (C) and TLR4(D). Data are mean ± SD. # p < 0.05, compared to normal control group; * p < 0.05, compared to CTX.

The IL-1β and TNF-α levels were significantly higher and the IL-10 level was significantly lower in the CTX group animals compared to the normal control group. Both doses of BAE significantly reversed the increase in IL-1β and TNF-α levels and the decrease in IL-10 level induced by CTX, with a dose-dependent manner (Fig 6A–6C, p< 0.05). Moreover, the expression of TLR4 protein was significant increase in CTX group compared with in the normal group and there was significant decreased of TLR4 protein in the both dose of BAE groups compared with the CTX group (Fig 6D, p< 0.05).

### BAE attenuated CTX-induced oxidative stress

**Fig 7:**
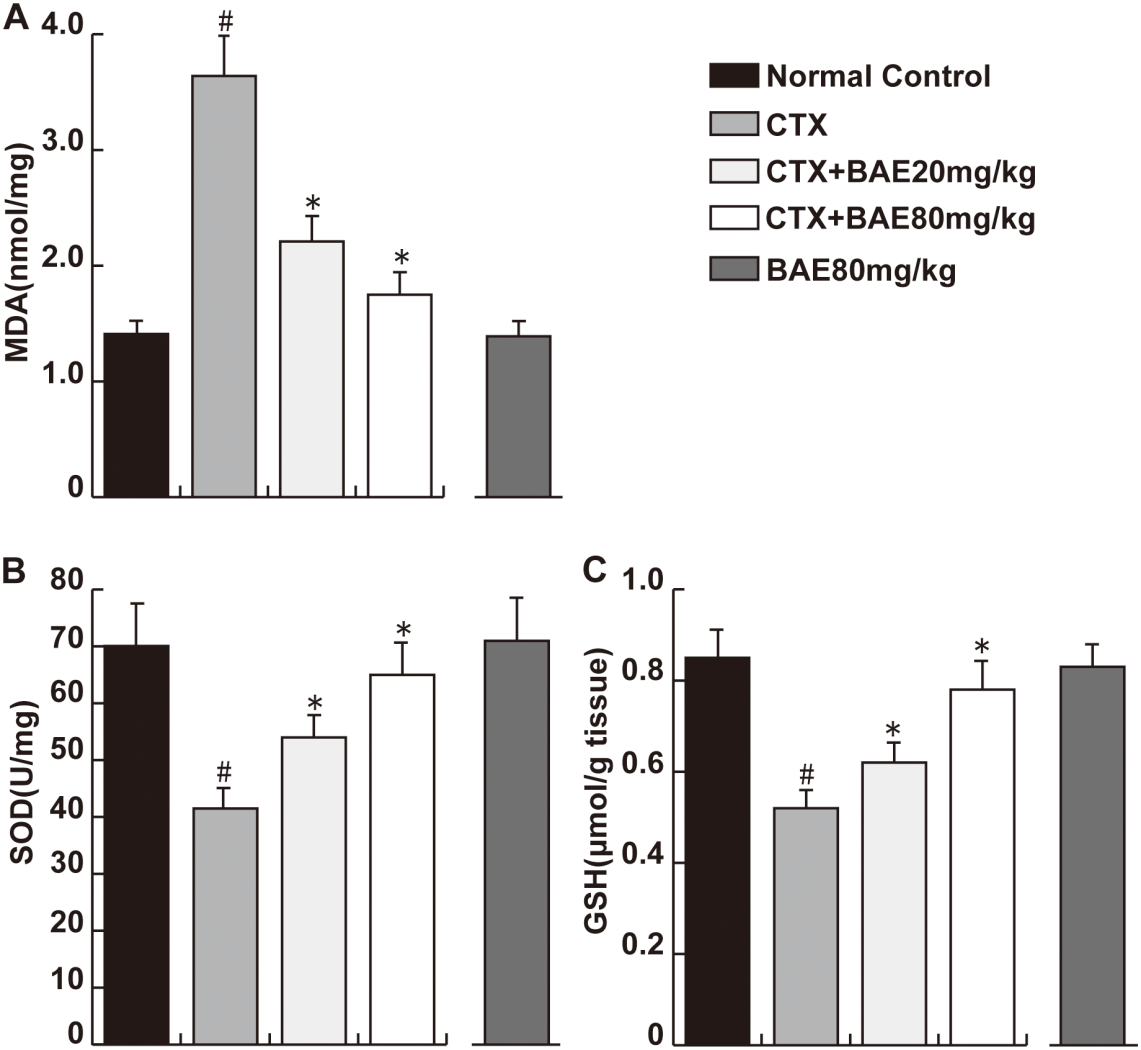
BAE attenuated CTX-induced oxidative stress. Heart tissues were performed to detect the levels of MDA(A), SOD(B) and GSH(C). Data are mean ± SD. ^#^ p < 0.05, compared to normal control group; * p < 0.05, compared to CTX.

To assess the effect of BAE on CTX-induced oxidative stress, we assessed MDA levels (a marker of oxidative stress), SOD and GSH activity (antioxidant status marker). The results demonstrated the highest MDA level in the CTX group among all the groups. 20 and 80 mg/kg BAE significantly inhibited the increase in MDA level. In addition, rats in the CTX group showed the lowest SOD and GSH activity, while BAE significantly antagonized the decrease in SOD and GSH activity induced by CTX (Fig 7A–7C, p< 0.05).

### BAE attenuated CTX-induced increase of Bax and decrease Bcl-2

**Fig 8:**
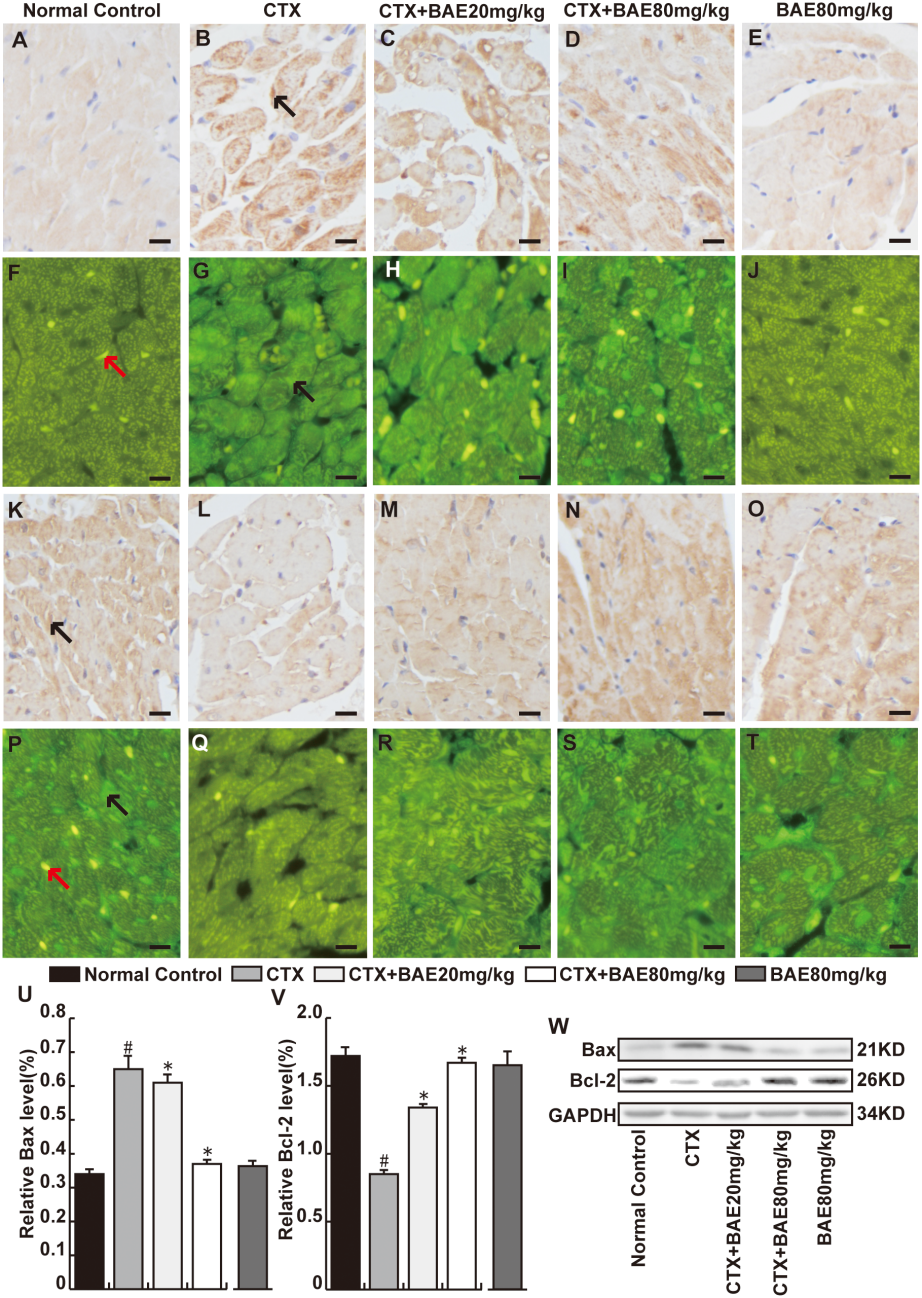
BAE attenuated CTX-induced cardiomyocyte apoptosis. The heart sections were reacted with anti-Bax (A-J) or anti-Bcl-2 (K-T) monoclonal antibody(immunohistochemical staining and immunofluorescence, Bar: 20 μm). We observed that the Bax in CTX group was more intense than normal control group in cytoplasm, whereas Bcl-2 was highly expressed in myocardial cells in normal control group was more intense than CTX group. The black arrow represents respectively the Bax or Bcl-2-positive cardiomyocytes and the red arrow represents the nuclear of cardiomyocytes. Both doses of BAE significantly decreased Bax and increased Bcl-2 proteins expression in cardiac tissues. (U-W)Western blot was performed to detect the Bcl-2 and Bax proteins expression. All proteins were normalized to the corresponding GAPDH. Right: western blot image. Left: statistic data. ^#^p < 0.05, compared to normal control; *p < 0.05, compared to CTX.

To investigate the effects of BAE on CTX-induced cardiomyocyte apoptosis, we detected established protein markers of cardiomyocyte apoptosis including Bax and Bcl-2 proteins using immunohistochemical staining, immunofluorescence and Western blot. There were significant increase in the number of Bax-positive cardiomyocyte and decrease in the number of Bcl-2 positive cell in CTX group compared with hearts in the normal group. However, both doses of BAE significantly attenuated cardiomyocyte apoptosis protein including Bax and Bcl-2 expression, with a dose-dependent manner (Fig 8A–8W, p< 0.05).

## Discussion

It is reported that high doses of CTX usually cause an acute cardiotoxicity within ten days of its administration. Cellular mechanisms of CTX-induced cardiotoxicity are considered to be mediated by an increase in free oxygen radicals (ROS), and inflammation and apoptosis events [4,26]. In the present study, we demonstrated that BAE attenuates CTX-induced decreased mean arterial blood pressure, increased heart rate and activities of heart enzymes, improved cardiac dysfunction, left ventricular hypertrophy and fibrosis. In addition, BAE also attenuated CTX-induced LV leukocyte infiltration and inflammatory cytokines expression, ameliorated oxidative stress reaction as well as cardiomyocyte apoptosis.

It is established that left ventricular hypertrophy is an independent and powerful predictor of heart failure and mortality [27]. Lung weight and LA weight are highly reliable markers for LV dysfunction. Increased evidence indicates that CTX, a cardiotoxic agent, leading to direct myocardial cells injury including cardiac hypertrophy, extensive necrosis with inflammatory cells infiltration [28,29]. In this study, we found that BAE attenuated significantly CTX-induced decreased blood and increased heart rate and reduced the heart weight/body, lung weight/body ratios and LA weight. Moreover, the results showed that increases in ANP, β-MHC and BNP mRNA expression, established gene markers of cardiac hypertrophy, were attenuated in the both dose of BAE group compared with the CTX group. ANP, a cardiovascular hormone, was mainly secreted in heart atria and was demonstrated protective effects on myocardial injury and cardiac hypertrophy [30]. BNP was secreted by the ventricles and was increased when ventricular dilation. BNP, ANP and β-MHC levels are not only elevated in asymptomatic left ventricular dysfunction patients, but also elevated in patients with heart failure [31,32]. Increased activities of heart enzymes including CK-MB, LDH and AST are well-known diagnostic indicators of cardiac injury, which are associated withmyocardial infarction, myocarditis and heart failure [33]. In this study, AST, LDH and CK-MB were significantly increased in the CTX group compared to the normal group. The administration of BAE showed dose dependent reduction in CTX-induced elevated biomarkers of cardiac injury. Importantly, Echo also demonstrated that BAE ameliorated significantly CTX-induced increased LVPW, LVEDd, LVESd, LVEDV, LVESV and decreased LVEF and LVFS. These observations support the conclusion that BAE attenuated CTX-induced cardiac hypertrophy and dysfunction.

Apoptosis is one of the major processes involed in heart failure, hypertrophy, and myocardial infarction [34]. Bax protein is a pro-apoptotic protein Bcl-2 family and it capable of directly triggering apoptosis. Bcl-2 is an critical anti-apoptotic protein and is activated in response to oxidative stress [35]. It was reported that CTX-induced cardiac toxicity is related to its ability to induce apoptosis in myocytes by different mechanisms [36]. In the present study, CTX resulted in a significant increase in Bax protein expression and a significant decrease in Bcl-2 protein expression in cardiac tissues. Both doses of BAE significantly significantly decreased Bax protein expression, and increased Bcl-2 protein expression in cardiac tissues, with a dose-dependent manner. It has been reported that BAE was able to restore the expression of apoptotic protein in UV-induced HepG2 [37]. Previous studies also demonstrated that BAE was beneficial to light-induced retinal pigment epithelial cells damage through the suppression of apoptosis [38]. Therefore, we speculated that BAE might have potential protective effect against CTX-induced cardiomyocyte apoptosis.

The left ventricle inflammation response is a common pathophysiological characteristic of the cardiovascular diseases, which contributes to the development and progression of multiple end-organ injuries. Several cytokines, including pro-inflammatory TNF-α and IL-1β, and anti-inflammatory IL-10, play an important role in heart inflammation. TNF-α is a crucial and is often released when toxic substances caused cardiac injury [39], which usually triggers cardiac dyfunction in acute heart injury [40]. IL-1β is produced by activated macrophages, and involved in a variety of cellular activities, including cell proliferation, differentiation, and apoptosis [41]. IL-10 is produced by T cells and macrophages, and is postulated to inhibit proinflammatory cytokine production, thereby preventing tissue damage [42]. A broad spectrum of in vitro and in vivo studies have shown that CTX could cause the inflammatoty response in various organs [43–45]. CTX has been reported to induce TNF-α and IL-1β [46,47], and reduce IL-10 expression [48]. The present study found that CTX produced massive change including cardiac hypertrophy, fibrosis, heart edema with inflammatory cell infiltration and the expression change of several cytokines such as IL-1β, TNF-α and promoting IL-10, which were antagonized by BAE. In fact, it has also been reported that BAE inhibited IL-1β and TNF-α expression, and promoted IL-10 secretion in cellular and animal models after inflammatory insults [49–51], which were consistent with our results, confirming the anti-inflammatory role of BAE. Berries have been studied widely for their antioxidant properties, however, few preclinical data was documented an important effects on inflammatory pathways [52]. Oxidative stress reflects an imbalance between the systemic manifestation of reactive oxygen species (ROS) and the ability of biological systems to readily detoxify the ROS or repair the resulting damage [53], which is usually evaluated by monitoring ROS-induced lipid peroxide (MDA) and antioxidative systems such as SOD and GSH expression [54]. In this study, we found that BAE attenuated CTX-induced increased MDA levels (a marker of oxidative stress), and decreased SOD and GSH activity (antioxidant status marker), which suggested that BAE have antioxidant properties. Moreover, TLR4, a member of pattern recognition receptors, is expressed in cells of myeloid lineage. Accumulated studies have shown that TLR4 plays an important role in the induction of the inflammatory response and is associated with tissue injury [55–57]. Recent evidence suggests that central blockade of TLR4 could improved myocardial inflammation and attenuated cardiac function in angiotensin II-induced hypertension [58]. It has been demonstrated that partially silencing brain TLR4 could prevent MI-induced LV remodeling in rats, suggested that brain TLR4 might to be a potential target of the heart failure [59]. We found that expression of TLR4 protein was significantly increased in CTX group compared with in the normal group and there was significant decreased of TLR4 protein in the both dose of BAE groups compared with the CTX group. All data above suggested that TLR4 protein might play an important role in the pathogenesis of CTX-induced LV inflammatory.

## Conclusions

BAE attenuates the CTX-induced cardiac injury and the protective mechanisms are related closely to the anti-inflammatory, antioxidant and anti-inflammatory characteristics of BAE.
